# The effect of Nifedipine on embryo transfer outcomes: A randomized clinical trial

**DOI:** 10.18502/ijrm.v20i12.12562

**Published:** 2023-01-09

**Authors:** Masoomeh Nataj Majd, Ashraf Moini, Saghar Samimi Sadeh, Ehsan Bastanhagh

**Affiliations:** ^1^Department of Anesthesiology, Arash Women's Hospital, Tehran University of Medical Sciences, Tehran, Iran.; ^2^Department of Obstetrics and Gynecology, Arash Women's Hospital, Tehran University of Medical Sciences, Tehran, Iran.; ^3^Breast Disease Research Center (BDRC), Tehran University of Medical Sciences, Tehran, Iran.; ^4^Department of Endocrinology and Female Infertility, Reproductive Biomedicine Research Center, Royan Institute for Reproductive Biomedicine, ACECR, Tehran, Iran.; ^5^Department of Anesthesiology, Yas Hospital, Tehran University of Medical Sciences, Tehran, Iran.

**Keywords:** Nifedipine, In vitro fertilization, Uterus, Contraction.

## Abstract

**Background:**

Endometrial receptivity is crucial for embryo implantation, and excessive uterine contraction reduces success. Nifedipine which is a calcium channel blocker, could decrease uterine contraction and improve pregnancy outcomes.

**Objective:**

This study aimed to assess the effect of Nifedipine before embryo transfer on the pregnancy outcome in women undergoing in vitro fertilization (IVF) in a tertiary center in Iran.

**Materials and Methods:**

150 women who were candidates for IVF were randomly assigned into 2 groups: group 1 received 20 mg Nifedipine 30 min before embryo transfer, and group 2 received no intervention. Blood pressure of the participants was monitored every 10 min for 1 hr under the supervision of an anesthesiologist. Finally, implantation rate and chemical and clinical pregnancy rates were compared between groups.

**Results:**

At the end of the study, 140 participants were included in the final analyses. No significant difference was observed in clinical pregnancy rates between groups (20% vs. 22%, p = 0.51)

**Conclusion:**

Nifedipine administration before embryo transfer does not improve the implantation and clinical pregnancy rates in women undergoing IVF.

## 1. Introduction

Infertility is characterized as the inability to conceive after one yr of unprotected intercourse, influencing nearly 20% of couples worldwide (1). Assisted reproduction technology, including in vitro fertilization (IVF) has helped many infertile couples to achieve pregnancies. One of the challenging issues affecting IVF success is embryo implantation (2). Implantation success depends on both embryo and endometrium readiness (3-5).

One of the limiting factors in the endometrium receptivity is excessive uterine contraction, which reduces the implantation rate (4-6) since it can push the embryo toward the fallopian tube or cervix/vagina (7). Excessive uterine contraction could also lead to ectopic pregnancy (8).

For controlling uterine contractions, cyclooxygenase inhibitors, progesterone, anti-inflammatories, β2-adrenoreceptor agonists, phosphodiesterase inhibitors, and antispasmodics are used (9-10). Calcium channel blockers, known medications for controlling blood pressure, can control the contractility of the smooth muscles and lead to uterine relaxation. Nifedipine is a calcium channel blocker that is safe during pregnancy. It causes uterine relaxation (11) without any side effects for the fetus (12), and its adverse effects on mothers are not severe, depending on its total dose (13).

A previous study in 2019 found that a single use of Nifedipine before embryo transfer had no major impact on the implantation rate or the clinical pregnancy rate (14).

This study aims to assess the effect of Nifedipine before embryo transfer on the pregnancy rate in women undergoing IVF in a tertiary center in Iran.

## 2. Materials and Methods

### Study design and participants

In this randomized clinical trial, 150 women were enrolled from September 2017 to July 2020 in the Arash hospital, Tehran, Iran. Our inclusion criteria were: IVF candidates women aged between 20 and 39 yr with a body mass index of 18-29 kg/m^2^; and the American Society Anesthesiologist physical status classification system I. All women with hypertension, hypotension, abnormal uterine cavity, contraindication for the use of estrogen, progesterone, and Nifedipine, the use of drugs interacted with cytochrome P450 activity including azole antifungals, cimetidine, cyclosporine, erythromycin, quinidine, terfenadine, warfarin, benzodiazepines, flecainide, imipramine, propafenone, and theophylline within 3 months before the study, serum follicle-stimulating hormone level > 20 mlU/ml on days 2-4 of the menstrual cycle, and irregular heartbeat were excluded.

### Sample size

According to the overall pregnancy rate of 13.6% and 36% in 2 group of control and intervention in the Sohrabvand study (15), and by considering 0.05 type I error and power of 0.8 and the possibility of 20% attrition in each group, the total sample sizewas calculated as 157 women, 78 in each group.

### Randomization

#### Sequence generation

We used balanced block randomization with computer-generated sequence in blocks of 6 to recruit subject in each arm. We used the ratio of 1:1 to allocate subjects in each arm.

Participants were randomly assigned into 2 groups (n = 75/each) using block randomization method. Block randomization was conducted using sealed envelope, and the randomization list was prepared by the statistician. In this study, the outcome assessors and our statistician who analyzed the data were blinded.

#### Allocation concealment mechanism

Mechanism used to implement the random allocation sequence (such as sequentially numbered containers), describing any steps taken to conceal the sequence until interventions were assigned. Then we put each of the random intervention according to the random sequence in to the envelope with a random code on it. Only the one who generates the sequence knows the intervention and placebo.

#### Blinding

A randomization list is prepared by the statistician. In this process, randomization control trial medicine was placed in similar packets. The sequence of medicine administration and the list of random allocation were not disclosed to dispensing practitioners. These packets were handed over to the dispensing nurse, who was unaware of the contents of each packet. When the doctor declares the eligibility of patients, the nurse then distributes the packets based on the identification numbering. Fulfillment of the final data is up to the individual who is unaware of the type of treatment.

### Interventions

All participants (n = 150) received the long gonadotropin-releasing hormone agonist protocol. On the 19-21 ${}^{\text{st}}$ day of the cycle before stimulation, gonadotropin-releasing hormone agonist (Cinnafact, Cinnagen, Iran) was started with subcutaneous injection at a dose of 0.5 mg/day for 10-14 days. Following menstrual bleeding, a transvaginal ultrasound assessment was performed. If the endometrium was thin or no follicles greater than 10-12 mm were detected, the controlled ovarian hyperstimulation cycle was started by daily subcutaneous injection of Cinnal-F (follitropin-alpha, 300 IU/ml, Cinnagen Inc, Tehran, Iran) and 75 IU of human menopausal gonadotropin (Pooyesh Darou Inc, Tehran, Iran). In case of no vaginal bleeding after 14 days of agonist injection, at first, a beta human chorionic gonadotropin pregnancy test was requested, and then, if the test was negative, Superfact injection was continued to achieve complete pituitary suppression. Gonadotrophin was continued until the development of a 17 mm sized dominant follicle. At this stage a 10,000 IU HCG ampule (Pooyesh Darou Inc, Tehran, Iran) was prescribed. The ovum-pick-up surgery was performed 35-36 hr after the human chorionic gonadotropin injection.

Group I received 20 mg oral Nifedipine (Tolid Daru, Tehran, Iran) 30 min before embryo transfer, and group II received no intervention.

Two physicians then grade the embryos as good or bad based on the criteria in Gardner and co-workers study (16). Embryos with grade 3AA, 4AA, 4AB, 4BA, 5AA, 5AB, 5BA, 6AA, 6AB, and 6BA were considered as good embryos for transfer in all cycles, fresh embryos transfer was scheduled.

All transfers were done by Cook catheter without Tenaculum and under ultrasound guide without any difficulties.

### Outcomes and data collection

In the present study, the clinical and chemical pregnancy rates were considered as the primary outcomes. Chemical pregnancy was checked by beta human chorionic gonadotropin test, 14 days after transfer. Clinical pregnancy was considered successful if a gestational sac was detected by ultrasound after 4 wk. Our secondary outcomes were the implantation rate, blood pressure variation, and multiple pregnancy rate. Blood pressure was monitored in all the participants in 3 intervals including at the time of anesthesia induction, end of anesthesia, and in recovery time, under the supervision of an anesthesiologist. The implantation rate was calculated as the number of gestational sacs divided by the number of embryos transferred to the uterus (17).

### Ethical considerations

All participants were asked to sign an informed consent form before the study. The study protocol was approved by the Ethics Committee of Tehran University of Medical Sciences, Tehran, Iran (Code: IR.TUMS.MEDICINE.REC.1395.1177P).

### Statistical analysis

Statistical package for the social sciences (SPSS) software version 13 was used for data analyses. The Fisher's exact test, Mann-Whitney, and Student's *t* tests were used to examine the differences between the 2 groups and compare the variables. A p-value < 0.05 was considered statistically significant.

## 3. Results

**Figure 1:**
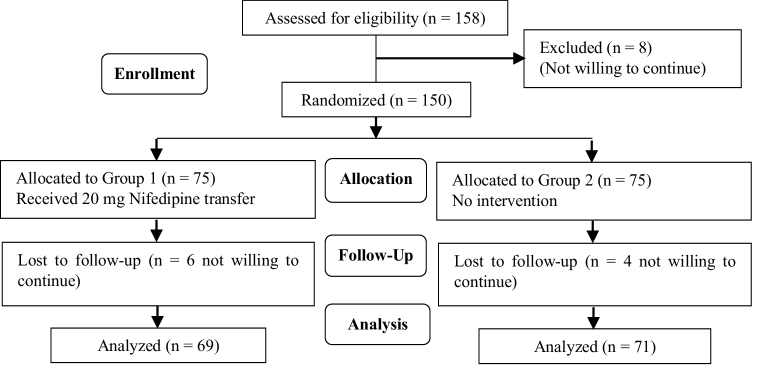
The study CONSORT flowchart.

In this study, 150 cases were randomly assigned into 2 groups. However, 6 patients from group I and 4 from group II withdrew from the study because they were not willing to continue. Finally, we analyzed 140 cases, 69 cases in group I and 71 cases in group II. The flow diagram of study is showed in figure 1.

No significant difference was observed in age, duration of infertility (Table I), retrieved oocytes, matured oocytes, and transferred embryos between the 2 groups (Table II).

However, no difference in blood pressure between the 2 groups was observed (Table III). No significant difference was observed in clinical and chemical pregnancy rates and multiple pregnancy between the 2 groups (Table IV).

**Table 1:** Basic characteristics of the participants in 2 study groups


**Variables**	**Group I (n = 69)**	**Group II (n = 71)**	**P-value**
**Age (yr)**	35.1 ± 4.9	34.9 ± 4.5	0.83
**Duration of infertility**	6.8 ± 3.9	7.7 ± 4.6	0.31
Data presented as Mean ± SD. Student's *t* test			

**Table 2:** Comparison of oocytes and transferred embryos between the 2 groups


**Variables**	Group I (n = 69) Median (IQR*)	Group II (n = 71) Median (IQR*)	**P-value**
**No. of retrieved oocytes**	7 (6.5)	8 (6)	0.51
**No. of matured oocytes**	6 (5)	6 (5)	0.50
**No. of transferred embryos**	2 (1.5)	2 (1)	0.82
Mann-Whitney U-test, *Interquartile range			

**Table 3:** Comparison of blood pressure between the groups


**Variables**	**Group I (n = 69)**	**Group II (n = 71)**	**P-value**
**SBP at anesthesia induction**	115.3 ± 6.9	113.1 ± 15.9	0.32
**SBP at the end of anesthesia**	119.2 ± 7.6	118.8 ± 7.7	0.72
**SBP at recovery**	114.6 ± 13.6	116.3 ± 7.9	0.32
**DBP at anesthesia induction**	66.5 ± 7.2	63.7 ± 6.9	0.24
**DBP at the end of anesthesia**	68.8 ± 6.8	66.9 ± 8.2	0.12
**DBP at recovery**	67.1 ± 6.5	65.4 ± 6.7	0.11
Data presented as Mean ± SD. Student's *t* test. DBP: Diastolic blood pressure, SBP: Systolic blood pressure			

**Table 4:** Comparison of pregnancy outcomes in the 2 study groups


**Pregnancy outcomes**	**Group I (n = 69)**	**Group II (n = 71)**	**P-value**
**Chemical pregnancy**	14	23	0.10*
**Clinical pregnancy**	14	16	0.51*
**Multiple pregnancy**	1	1	0.50**
Data presented as n (%). *Chi-square test, **Fisher's exact test			

## 4. Discussion

This study aimed to assess the effect of Nifedipine before embryo transfer on the pregnancy rate in women undergoing IVF. To the best of our knowledge, this is the first study evaluating the effects of Nifedipine on pregnancy outcomes in women undergoing IVF in Iran. The results showed that pregnancy outcomes such as clinical pregnancy, multiple pregnancies, and implantation rates were not significantly different between the groups.

There are numerous methods to improve the quality of the transfer as a final step of the assisted reproductive technique, like ultrasound guidance, soft embryo transfer, and some pharmacological agent (18). There are different pharmacological approaches to improve IVF outcome, such as the addition of growth hormone or growth hormone releasing factor, oral L-arginine, transdermal testosterone, letrozole, Nifedipine (14), platelet-rich plasma (19), and granulocyte-macrophage colony-stimulating factor (20).

Excessive uterine contraction is one of the main causes of the pregnancy failure in women undergoing IVF, in line with this concept and the role of the antispasmodic agents for the uterine receptivity. Several studies about the niphedipine, hyoscine, and anti-inflammatory agents, have been performed (9, 10). One study showed that Piroxicam does not have a significant effect on the pregnancy rate (21). Atosiban, an oxytocin/vasopressin receptor antagonist, is another agent that decreases uterine contraction by reducing prostaglandin production. It also improves the uterine blood supply (22). A meta-analysis showed that the administration of atosiban on embryo transfer day will improve the implantation rate by 92% (9).

Nifedipine, used in gynecology as a tocolytic agent, has vasodilatory and uterine relaxation effects (11). According to prior studies, Nifedipine has little effect on uterine perfusion, and maternal blood pressure (12, 13).

In a clinical trial in 2019, which compared 46 IVF candidates in the Nifedipine group (30 min before embryo transfer) with 47 candidates in the placebo group, no significant difference was found regarding implantation rate, multiple pregnancy rate, and clinical pregnancy between groups (14).

This study had some limitations. First, we did not use a placebo for the control group, so there was no blinding. Second, the study was a single-center study. Hence, large multicentric, placebo-controlled studies are recommended.

## 5. Conclusion

The finding of this study showed that Nifedipine administration before embryo transfer does not improve the implantation and clinical pregnancy rates in women undergoing IVF. Clinical trial studies with large sample size was recommended.

##  Conflicts of Interest

The authors have no conflict of interest.
